# A temporal examination of calcium signaling in cancer- from tumorigenesis, to immune evasion, and metastasis

**DOI:** 10.1186/s13578-018-0223-5

**Published:** 2018-04-03

**Authors:** MengMeng Xu, Andreas Seas, Musa Kiyani, Keven S. Y. Ji, Hannah N. Bell

**Affiliations:** 1 Medical-Scientist Training Program, Duke University Medical Center, Durham, NC 27710 USA; 2 Department of Pharmacology and Cancer Biology, Duke University Medical Center, Durham, NC 27710 USA; 3 School of Medicine, Duke University Medical Center, Durham, NC 27710 USA; 4 Duke-NUS Medical School, Singapore, 169857 Singapore

**Keywords:** Calcium signaling, Cancer, Immortalization, Tumor-stromal interaction, Metastasis, Drug resistance

## Abstract

**Background:**

Although the study of calcium (Ca^2+^) is classically associated with excitable cells such as myocytes or neurons, the ubiquity of this essential element in all cellular processes has led to interest in other cell types. The importance of Ca^2+^ to apoptosis, cell signaling, and immune activation is of special import in cancer.

**Main:**

Here we review the current understanding of Ca^2+^ in each of these processes vital to the initiation, spread, and drug resistance of malignancies. We describe the involvement of Ca^2+^, and Ca^2+^ related proteins in cell cycle checkpoints and Ca^2+^ dependent apoptosis and discuss their roles in cellular immortalization. The role of Ca^2+^ in inter-cellular communication is also discussed in relevance to tumor-stromal communication, angiogenesis, and tumor microinvasion. The role that Ca^2+^ plays in immune surveillance and evasion is also addressed. Finally, we discuss the possibility of targeting Ca^2+^ singling to address the most pressing topics of cancer treatment: metastatic disease and drug resistance.

**Conclusion:**

This review discusses the current understanding of Ca^2+^ in cancer. By addressing Ca^2+^ facilitated angiogenesis, immune evasion, metastasis, and drug resistance, we anticipate future avenues for development of Ca^2+^ as a nexus of therapy.

## Background

Investigational interest in calcium (Ca^2+^) began over 100 years ago with the discovery of the requirement for Ca^2+^ in the contraction of rat cardiac muscle [1]. Due to this initial discovery, Ca^2+^ was thoroughly characterized in ventricular action potential and other muscle cell types before the same basic principles were applied to other excitatory cells types, such as neuronal cells [2]. The importance of active zone localized Ca^2+^ channels to neurotransmitter release further reinforced the importance of Ca^2+^ in proper cell function. Today, Ca^2+^ is known to be an essential element vital to the health and function of every cell type. Amplification in the magnitude and duration of Ca^2+^ changes in the cytosol could mean the difference between cellular migration and cell death [3, 4]. Similarly, increases in mitochondrial Ca^2+^ can signal either increased ATP synthesis or trigger cell death [5]. This fine control of cytosolic and organelle Ca^2+^ levels relies on an intricate symphony between a wide variety of Ca^2+^ channels pumps and exchangers [2]. In this review, we provide an overview of how disruptions in Ca^2+^ regulation affect cancer progression, from its involvement in the immortalization of tumor cells, to its role in tumor-stromal interactions and epithelial–mesenchymal transition, and finally to current research on Ca^2+^ in drug resistance.

### Role of intracellular Ca^2+^ in cell cycle and death

**Fig. 1:**
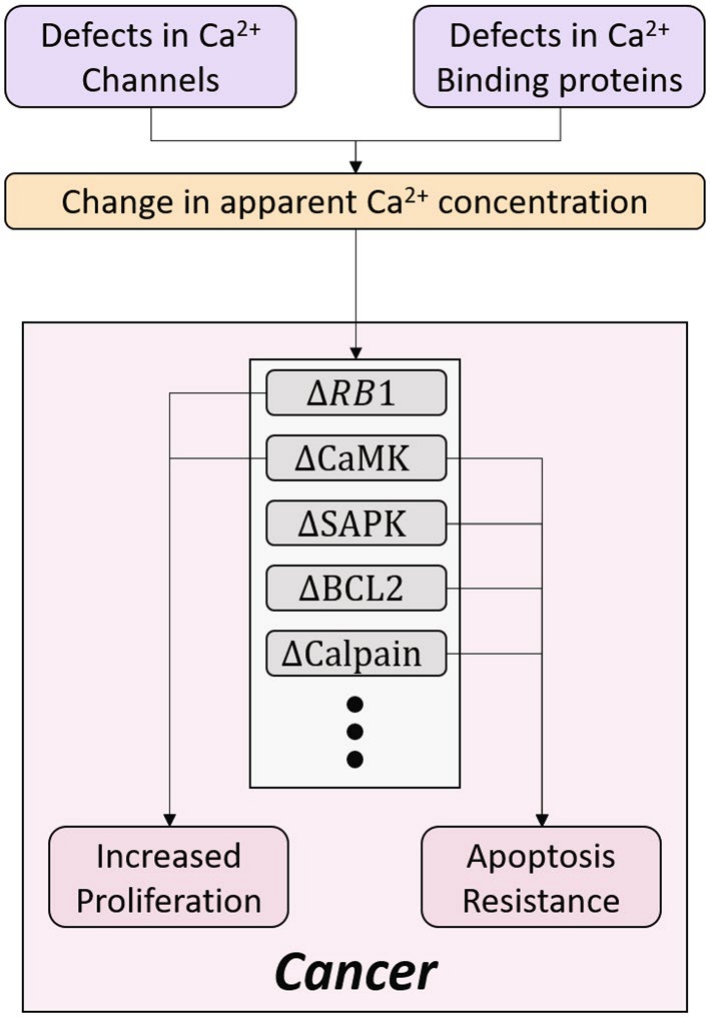
Ca^2+^ and associated protein involvement in cellular proliferation. The affect of Ca^2+^ concentration on key cellular proteins are diagrammed

Given the greater than ten-fold gradient between cytosolic (~ 100 nM) and extracellular (> 1 mM) Ca^2+^ levels, opening of intramembrane Ca^2+^ channels leads to an immediate influx of Ca^2+^ [1]. Upon reaching the cytoplasm, Ca^2+^ often forms complexes with calmodulin to regulate a variety of kinases and cyclins, which regulate cell proliferation and apoptosis [6, 7]. Ca^2+^ regulates global cellular processes in such a way that any disturbances to Ca^2+^ homeostasis via alterations in expression or folding of Ca^2+^ channels and Ca^2+^ binding proteins can disrupt the cell-cycle [8]. As a result, dysregulation of intracellular Ca^2+^ levels can affect the ability of cells to regulate progression through the cell-cycle and lead to unchecked proliferation and tumorigenesis [9], two of the ten hallmarks of cancer (Fig. 1).

**Fig. 2:**
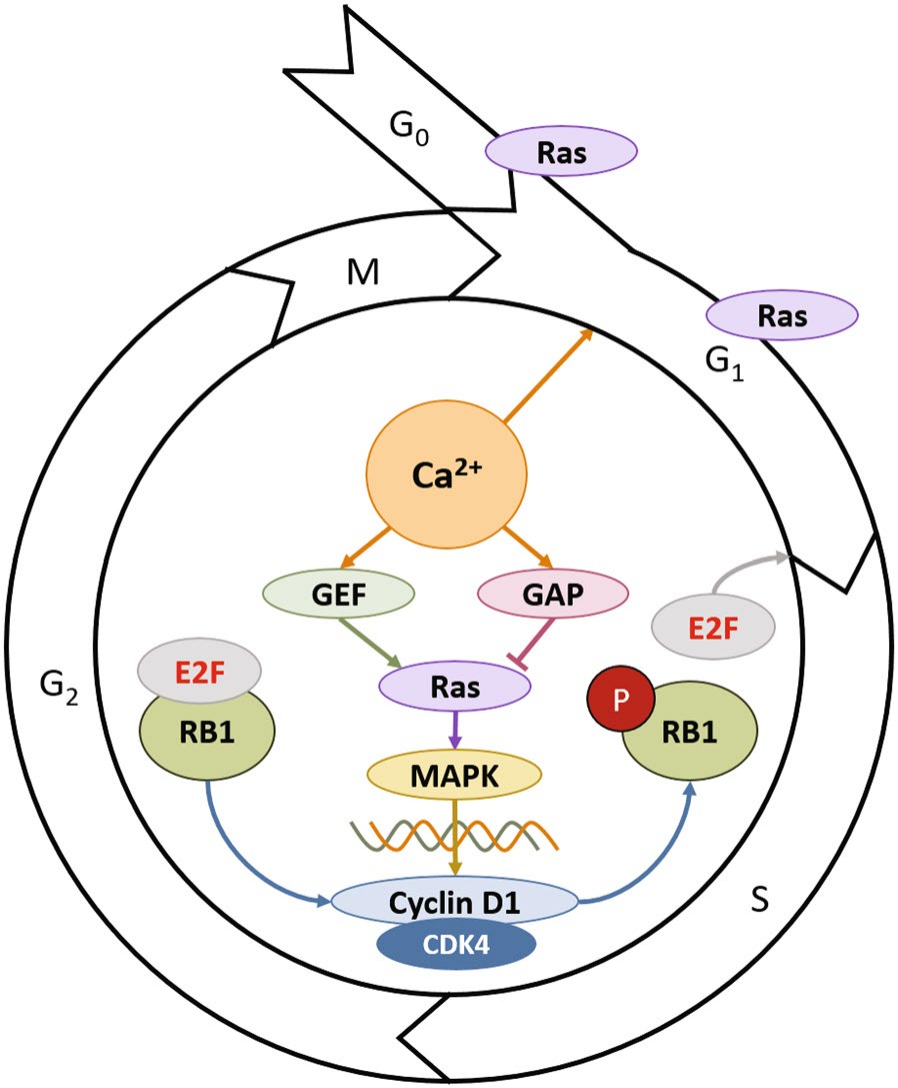
Schematic of cell cycle, and the influence of calcium in G1/S transitioning via the MAPK pathway. Note that Ras, a protein under control of cytosolic calcium levels, also regulates the G0/G1 transition, and is important throughout G1 phase

In the normal cell, progression from G1 to S-phase is accomplished via phosphorylation and subsequent inactivation of the tumor suppressor, RetinoBlastoma protein 1 (RB1), as illustrated in Fig. 2 [10]. Endogenous RB1 inactivation or deletion removes this check on the cell cycle and allows affected cells to undergo unchecked DNA synthesis, leading to an accumulation of potentially oncogenic DNA damage. Normally, cytosolic Ca^2+^ levels modulate the activity of guanosine exchange factor (GEF), a Ras stimulator, and GTPase activating protein (GAP), a Ras inhibitor. When activated, Ras stimulates the proliferative mitogen-activated protein kinase (MAPK) pathway, which results in upregulation of cyclin D1 in the cytoplasm, with ultimate phosphorylation of RB1 and release of the E2F transcription factor which initiates the cells transition into S-phase (Fig. 2). This connection between calcium and RB1 indicates that increased cytosolic Ca^2+^ levels can lead to constitutive activation of the MAPK pathway, causing removal of the G1-S transition check point. Ca^2+^ is also involved in signaling entry into G1, as well as the transition from G2 to M, although the mechanisms of its involvement at these check points are not well understood [11].

Other cell cycle related families, like the Ca^2+^/calmodulin-dependent protein kinases (CaMKs) are also known to facilitate proliferation and avoid death by promoting passage through the cell cycle and resisting apoptotic mechanisms [12]. CaMK levels have been shown to vary in lymphoma, ovarian cancer, and hepatocellular carcinoma, among others [13–15].

### Changes in Ca^2+^ conduction and levels can lead to apoptosis evasion and immortalization

In normal tissue, large, sustained changes to cytosolic Ca^2+^ can initiate cell death. Ca^2+^ flux from the endoplasmic reticulum (ER) to the mitochondria can also result in increased mitochondrial sensitivity to apoptotic stimuli. Chronic Ca^2+^ depletion is also known to cause ER stress and activation of stress activated protein kinases (SAPKs), which leads to apoptosis [11]. Finally, high cytosolic levels of Ca^2+^ can lead to cell death by activating calpain, a cysteine protease that specifically lyses BCL2, an anti-apoptotic regulatory protein [16, 17]. Alterations in Ca^2+^ levels can help cancer cells evade the first of these pathways by interrupting the transfer of Ca^2+^ from the ER to the mitochondria. Specifically, Ca^2+^-permeable Inositol 1,4,5-triphosphate receptor (IP3R) channels that facilitate this pro-apoptotic flux of Ca^2+^ from the ER could be prevented form activating. This process is aided by the anti-apoptotic capabilities of BCL-2, which diminishes Ca^2+^ flux by binding IP3Rs or decreasing Ca^2+^ levels in the ER lumen [18, 19]. Certain cancer types are also known to regulate cytosolic Ca^2+^ to their advantage by bleeding off excess Ca^2+^ to create pro survival conditions. This is evident in breast cancer, where over-expression of plasma membrane calcium-ATPase 2 (PMCA2) allows for the release of Ca^2+^ in conditions of Ca^2+^ overload [20]. Potential therapeutics blocking BCL2 activation, promoting stability of the ER-mitochondrial linkage, or blocking the PMCA2 “emergency-release valve” could induce Ca^2+^-triggered apoptosis in tumor cells.

### Tissue pressure, hypoxia and H^+^ can elicit Ca^2+^ changes

The cancer microenvironment consists of two interactive components: neoplastic cells and stroma [21]. The tumor stroma is a complex environment consisting of a non-cellular extracellular matrix (ECM), and fibroblasts, epithelial, endothelial, and immune cells [22]. This stroma is responsible for providing the nutrients, O_2_, and signaling molecules necessary to support tumor growth. In pancreatic adenocarcinoma, transient receptor potential cation channel 1 and 6 (TRPC1 and TRPC6) are activated by elevated pressure and hypoxia, respectively. This process also leads to Ca^2+^ entry and subsequent pro-angiogenic signaling cascade [23, 24]. In hepatocellular cancer cells, hypoxia also activates an ER Ca^2+^ sensor, stromal interaction molecule 1 (STIM1), which mediates activation of store-operated Ca^2+^ entry (SOCE) and leads to upregulation of hypoxia-inducible factor 1 (HIF-1) expression [25, 26]. HIF-1 then promotes release of growth factors (GFs) such as angiopoietin 2, placental GF, and stromal-derived factor 1 to promote angiogenesis [27]. In breast cancer, acid-sensing ion channel 1 (ASIC1) mediates Ca^2+^ influx. This pathway promotes tumor progression by forming reactive oxidative species and nuclear factor kB (NF-kB). Silencing ASIC1 has been shown to reduce tumor growth and metastasis in xenograft models [28]. Similarly, in pancreatic cancer cells, ASIC1 and ASIC3 mediate acidity-induced Ca^2+^ influx to promote epithelial–mesenchymal transition. Indeed, knockdown of ASIC1 and ASIC3 has been confirmed to suppress liver and lung metastasis in xenograft models.

### Ca^2+^-dependent tumor-stromal signaling drives angiogenesis

Communication between tumor and stromal cells has been shown to maintain growth and expansion through Ca^2+^-dependent signaling [29]. Vascular endothelial growth factor (VEGF) released by tumor cells triggers signal transduction that facilitates Ca^2+^-activated proliferation in endothelial cells. Upon VEGF receptor 2 activation, phosphoinositide phospholipase C (PLCγ) is phosphorylated, which in turn hydrolyzes phospholipid phosphatidylinositol (4,5)-bisphosphate (PIP_2_), resulting in accumulation of diacylglycerol (DAG) and inositol 1,4,5-trisphosphate (IP_3_). Accumulation of IP_3_ results in increase of intracellular Ca^2+^ and activation of the proliferative MAPK pathway [30, 31]. Proliferation in numerous subtypes of breast and gastrointestinal carcinomas, and glioblastomas is dependent upon this process [32–34]. Similarly, basic fibroblast growth factor (BFGF) activates the transient receptor potential cation channel subfamily V member 4 (TRPV4) in endothelial cells to facilitate Ca^2+^ influx, leading to endothelial cell proliferation, migration and angiogenesis [35, 36].

### Ca^2+^-dependent signaling may promote or hinder tumor escape of immune surveillance

**Fig. 3:**
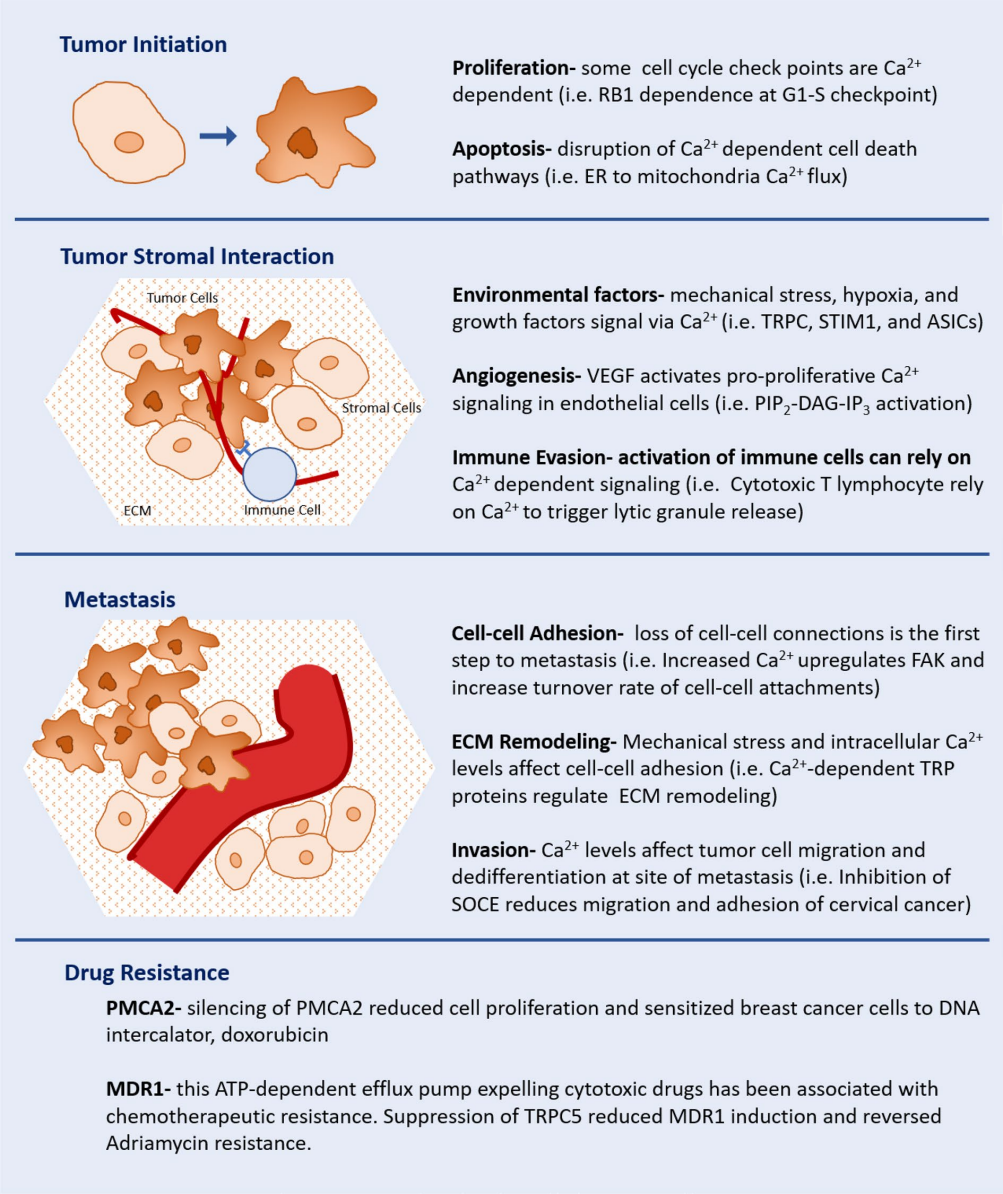
Ca^2+^ signaling in tumor progression. The involvement of Ca^2+^ in every step of tumor development, metastasis, and current knowledge on Ca^2+^ facilitated drug resistance

Ca^2+^ dependent signaling is critical in the functioning of tumor-associated macrophages (TAMs), which have the ability to both sustain tumor growth and exert anti-tumor effects under certain conditions [37]. TAMs induce tumor progression through chemokine ligand 18 (CCL18) production. In breast cancer, CCL18 binds to phosphatidylinositol transfer protein membrane-associated 3 (PITPNM3) at the plasma membrane and induces phosphorylation of PLCγ1 and protein kinase C zeta (PKCζ). This cascade increases levels of inositol 1,4,5-triphosphate 3-kinase isoform B (IP3KB), which are mediators in the Ca^2+^ signaling pathway. Indeed, the expression of CCL18 in blood or cancer stroma is associated with metastasis and reduced survival [38]. On the other hand, when T cell receptors (TCRs) on cytotoxic T lymphocytes binds to MHC-antigen receptors on a malignant cell, the resultant immune synapse triggers Ca^2+^ influx in the immune cell, leading to lytic granule release and tumor killing. TCR stimulation can also evoke Ca^2+^ release from the ER via signaling cascade involving Zeta-chain-associated protein kinase 70 (ZAP-70), lymphocyte-specific protein tyrosine kinase (Lck), linker of activation of T cells (LAT), PLC-γ, and IP3 [39, 40]. Similarly, Ca^2+^ entry through Orai1 channels is required for release of lytic granules and subsequent tumor cell destruction by natural killer cells [41]. Lastly, recent experiments with chimeric antigen receptor T (CAR T) cells, which have faster release-rates from dying tumor cells than T cell receptor (TCR) T cells do, have implied there is no difference in intensity of Ca^2+^ flux between the two cell types; therefore both trigger the release of tumor-killing particles at the same threshold level of Ca^2+^ [42]. Interactions between various components of stroma and tumor are shown in Fig. 3.

### Developing areas in tumor stromal Ca^2+^-dependent signaling

Recent findings on transient receptor potential cation channel subfamily A member 1 (TRPA1) and secreted protein acidic and rich in cysteine (SPARC) point to areas in need of further exploration. In prostate cancer stromal cells, TRPA1 has been shown to act as a mechanosensor and have the ability to bind to Triclosan, an antibacterial agent [43]. This binding increases Ca^2+^ in stromal cells to trigger subsequent secretion of mitogenic factors, which lead to proliferation and/or migration of adjacent epithelial and endothelial cells to promote angiogenesis [21]. However, the specific stromal ligand activating this function has yet to be discovered. SPARC, a multifunctional, matricellular Ca^2+^ binding protein, overexpressed in glioblastoma and thyroid, esophageal, hepatocellular, and pancreatic carcinomas, has been clinically correlated with tumor progression [44–47]. SPARC contains an N-terminal low-affinity Ca^2+^-binding domain and a C-terminal high-affinity Ca^2+^-binding domain [48]. This protein plays a crucial role in cell rounding and focal adhesion disassembly during angiogenesis, tumor invasion, and metastasis [49]. While the prevalence of Ca^2+^ binding domains in this protein hint at a role in SPARC function, the exact pathway through which a Ca^2+^-SPARC complex elicits tumor advancement remains largely unknown [50]. The continued mystery surrounding the mechanism of Ca^2+^ associated TRPA1 and SPARC function identifies the needs to continued investigation into Ca^2+^-dependent signaling in the tumor stroma.

### Impact of Ca^2+^ signaling on the epithelial–mesenchymal transition

The first step in metastasis is the loss of cell–cell connections. Focal adhesion kinase (FAK) is a ubiquitously expressed cytoplasmic tyrosine kinase that increases turnover of cell–cell contacts [51]. Overexpression of FAK is commonly associated with cancer, and seems to induce resistance to anoikis, death due to loss of attachment to a basement membrane. Increased intracellular Ca^2+^ upregulates FAK at focal adhesions through phosphorylation by the calmodulin-dependent protein kinase II (CaMKII) [52]. Thus, aberrant signaling resulting in elevated intracellular Ca^2+^ levels can lead to an increase in FAK and a higher turnover rate for cell–cell attachments [53]. Calcineurin, a protein regulated by Ca^2+^, recycles integrins in migrating cells and is another potential mediator of Ca^2+^-induced migration [54]. Except for this dysregulation of Ca^2+^, there are currently no other known differences between normal and malignant cells capable of migration [55].

Mechanical stress and intracellular Ca^2+^ levels affect cell–cell adhesion through TRP family proteins [56]. In addition to the above described role of TRP in cellular proliferation, TRP also plays a role in the epithelial–mesenchymal transition. High TRP levels are associated with the loss of cell adhesion, while TRP loss is associated with increased strength and number of focal adhesions [57]. Higher expression of TRP family member TRPV1 has been associated with increased migration in many different cancer cell lines [58, 59]. TRPV2 has also been shown to be an important regulator of matrix metalloproteases MMP2 and MMP9, which are required for the extensive ECM remodeling necessary for successful metastasis [60]. ECM remodeling enzymes are substantially upregulated or specifically induced in many cancers [61]. In addition, many ECM proteins themselves are controlled by calcium levels in the cell. From the glycoprotein fibrinogen which has multiple calcium binding sites critical for structure and function to fibrillin, which has several calcium binding epidermal growth factor domains to the thrombospondins which have multiple calcium binding repeats, calcium is a crucial player in normal physiology of the extracellular matrix. The overall effect of Ca^2+^ on ECM maintenance and remodeling remains an unanswered question, and an active area of research.

The epithelial–mesenchymal transition (EMT) is also associated with an increased capacity for invasion. This invasive capability has been connected to Ca^2+^ signaling in some cell types [62]. Davis et al. [63] have shown that when EMT is induced, there is an increase in cytosolic Ca^2+^ levels in human breast cancer cells. Chelating Ca^2+^ in this instance reduced epidermal growth factor levels and blocked the induction of EMT markers. Another important contributor to proliferative ability is the SOCE system, through which Ca^2+^ is pumped into the cytosol when ER Ca^2+^ is depleted. SOCE inhibitors have been shown to inhibit migration of cervical cancer and reduce the association of focal adhesion kinases at focal adhesion sites [62].

Extracellular Ca^2+^ levels have also demonstrated an effect on the re-differentiation of epithelial breast cancer lines. Re-differentiation after metastasis, is important in allowing cancer to survive in a novel niche after metastasis. Although physiological levels of Ca^2+^ inhibit proliferation and invasion, higher than normal extracellular levels increase estrogen receptor activity, which has been associated with more aggressive and invasive breast cancers [64]. High extracellular Ca^2+^ levels ultimately increase the risk of bony metastasis in both breast and prostate cancer [65].

### Targeting Ca^2+^ as a treatment modality for metastatic disease

Tumor metastases cause the majority of cancer deaths. As such, development of preventative measures against and treatment of metastasis is an extremely active area of research. Metastatic transformation requires the loss of epithelial cell–cell connections and the transformation of primary tumor cells into a migratory mesenchymal cell. During this process, the cells must also degrade the ECM, cross basement membranes, and enter the circulatory system. As detailed above, Ca^2+^ signaling is involved in every step of this process [66–68]. Therapeutically, targeting Ca^2+^ signaling to prevent metastasis is challenging, as any inhibition is likely to impact normal cells as well. Coupling Ca^2+^ to a cancer specific target has been shown to reduce normal cell death in a prostate cancer study [69]. For example, a drug combining Thapsigargin, a sarcolemma and ER Ca^2+^-ATPase (SERCA) inhibitor, with the targeting peptide for a prostate-specific antigen was able to limit cell death to prostate cancer cells while sparing normal cells [70]. Despite such technological advances, Ca^2+^-dependent migration mechanisms between normal and cancerous cells are similar enough that another mode of targeting Ca^2+^ should be considered [71]. As we have learned from “undruggable” proteins like Ras and Myc, targeting downstream effectors of Ca^2+^-dependent signaling, such as proteins associated with cell–cell contacts and ECM degradation, may be a more practical approach [72].

### Alterations in Ca^2+^ signaling in settings of drug resistance

In addition to being implicated in the described processes of tumor progression, Ca^2+^ might also play a significant role in facilitating drug resistance. In a recent study on breast cancer cell lines, increased mRNA levels of plasmalemmal Ca^2+^ efflux pump (PMCA2), which removes Ca^2+^ from the cell, was correlated with poor survival [73]. Silencing of PMCA2 reduced cell proliferation and sensitized these cells to doxorubicin. Elevated PMCA2 is commonly found in the mammary glands of lactating mice and may thus indicate high cellular metabolic activity, which is also frequently found in malignant cells. High levels of PMCA2 have also been confirmed in a variety of breast cancer cell lines. Another study confirmed the relationship between high PMCA2 expression and poor outcome, and demonstrated the ability of PMCA2 suppression to sensitize mammary epithelial cells to apoptosis [74].

*P*-glycoprotein or multidrug resistance protein 1 (MDR1), an ATP-dependent efflux pump that expels cytotoxic drugs, has also been associated with chemotherapeutic resistance in breast cancer [75]. Induction of this protein has been associated with upregulation of the Ca^2+^-permeable channel TRPC5 in adriamycin-resistant breast cancer cell lines. In both human and mice models, TRPC5 expression is often higher in tumor cells and concentrated to vesicles. Indeed, in the adriamycin-resistant breast cancer study, suppressing the activity of pro-oncotic TRPC5 reduced MDR1 induction and reversed adriamycin resistance both in vitro and in vivo [73]. TRPC5 suppression also appears to be essential to drug resistance in colorectal cancer, where suppression of TRPC5 expression reduced MDR1 induction, leading to 5-FU resistance via the canonical Wnt/β-catenin signal pathway.

A subtype of TRPC6 has also been implicated in another malignancy infamously recalcitrant to multiple chemotherapeutic regimens, hepatocellular carcinoma (HCC). A recent study has shown that a subtype of TRPC6, usually expressed at low levels in normal hepatocytes, mediates Ca^2+^ signaling and drug resistance in HCC. In this study, inhibition of Ca^2+^ signaling via TRPC6 inhibition resulted in restored sensitivity of HCC cells to various chemotherapeutic drugs and attenuation of the epithelial–mesenchymal transition [76]. These in vitro studies were further corroborated in xenograft models where TRPC6 inhibition enhanced doxorubicin efficacy. The same study also identified the STAT3 pathway as the mechanism of action for TRPC6/Ca^2+^ mediated drug sensitivity. Namely, reduction of intracellular Ca^2+^ via TRPC6 inhibition activates STAT3, which then stimulates re-differentiation of cells and restores drug sensitivity [77]. T-type Ca^2+^ channels have also been associated with drug resistance in ovarian and other high-morbidity gynecological malignancies. Experiments on mice models of ovarian cancer have shown mibefradil inhibition of T-type Ca^2+^ channels to sensitize the disease to carboplatin. Furthermore, both pharmaceutical and genetic inhibition of Ca^2+^ channels led to apoptotic growth suppression in the ovarian cancer cells [78].

Drug resistance, especially the development of multi-drug resistant disease, is of special concern in cancer therapy. The fact that Ca^2+^-mediated signaling can restore drug sensitivity in breast, colorectal, hepatocellular, and ovarian cancers suggests a possible role for Ca^2+^ channel blockers as an adjuvant therapy to standard-of-care chemotherapies.

## Conclusions

From tumor initiation to metastasis and drug resistance, Ca^2+^ signaling is intrinsic to all aspects of cancer biology (Fig. 3). Ironically, the very ubiquity of Ca^2+^ signaling in cancer makes this essential element difficult to explore in detail and target for drug development. While multiple studies have shown the importance of Ca^2+^ signaling at every key disease turning point (immortalization, metastasis, and drug response), isolation of the specific effects remains elusive. This suggests development of therapies targeting Ca^2+^ should be constructed using experience from other “undruggable” targets like Ras and Myc. Instead of targeting Ca^2+^ itself, known Ca^2+^ associated proteins such as PMCA2, TRPC5, and MDR1 may serve as more discerning objectives.

Another emerging field of interest for Ca^2+^ signaling is immunotherapy. Recent publications have suggested that calcium signaling could be used to improve the efficiency of immunotherapy approaches by enhancing antigen presentation and in the adaptive immune response. In addition, the role of Ca^2+^ in killing by natural killer cells and cytotoxic T lymphocytes may also be exploited as high levels of intracellular Ca^2+^ are required for efficient cancer cell killing activity. Conversely, Ca^2+^ reduction has been showing to reduce growth of malignant cells themselves. Thus, it is necessary to identify the specific Ca^2+^ channels utilized in granule exocytosis so that immune system’s ability to kill malignant cells can be enhanced without simultaneously promoting tumor growth. Although immunotherapy is a promising field through which Ca^2+^ signaling could augment treatment efficacy, the ubiquity of Ca^2+^ in normal metabolism and cellular function makes greater understanding of specific mechanisms in Ca^2+^ signaling necessary before such dreams become attainable.

## Abbreviations

ASIC1: acid-sensing ion channel 1
BFGF: basic fibroblast growth factor
CaMKII: Ca^2+^/calmodulin-dependent protein kinase II
CaMK: Ca^2+^/calmodulin-dependent protein kinases
CCL18: chemokine ligand 18
DAG: diacylglycerol
ECM: extracellular matrix
EMT: epithelial–mesenchymal transition
ER: endoplasmic reticulum
FAK: focal adhesion kinase
HIF-1: hypoxia-inducible factor 1
IP3: inositol 1,4,5-trisphosphate
IP3KB: inositol 1,4,5-triphosphate 3-kinase isoform B
IP3R: 1,4,5-triphosphate receptor
LAT: linker of activation of T cells
Lck: lymphocyte-specific protein tyrosine kinase
MAPK: mitogen-activated protein kinase
MDR1: *p*-glycoprotein or multidrug resistance protein 1
NF-kB: nuclear factor kB
PIP2: phospholipid phosphatidylinositol (4,5)-bisphosphate
PITPNM3: phosphatidylinositol transfer protein membrane-associated 3
PKCζ: protein kinase C zeta
PLCγ: phosphoinositide phospholipase C
PMCA2: plasma membrane calcium-ATPase 2, plasmalemmal Ca^2+^ efflux pump
RB1: retinoblastoma protein 1
SAPK: stress activated protein kinases
SERCA: sarcolemma and ER Ca^2+^-ATPase
SOCE: store-operated Ca^2+^ entry
SPARC: secreted protein acidic and rich in cysteine
STIM1: stromal interaction molecule 1
TAMs: tumor-associated macrophages
TCRs: T cell receptors
TRPA1: transient receptor potential cation channel subfamily A member 1
TRPC: transient receptor potential cation channel
TRPV4: transient receptor potential cation channel subfamily V member 4
VEGF: vascular endothelial growth factor
ZAP-70: zeta-chain-associated protein kinase 70
